# Supplementary material for: Beyond health: A machine learning analysis of structural barriers to school attainment in Somalia
Source: PLoS One. 2025 Oct 16;20(10):e0326958. doi: 10.1371/journal.pone.0326958 (PMC12530525; doi:10.1371/journal.pone.0326958)
Supplement: Attachment — Submitted filename: Response to Reviewers 20250720.docx [file pone.0326958.s003.docx]

Hodo Abdikarim

Research and Innovation Centre, Amoud University, Borama, Somaliland

20 July 2025

**The Editorial Team**

**PLOS ONE**

**Subject: Response to Reviewers for Manuscript ID [PONE-D-25-31078] - [EMID:b8d04c918202b6bd]**

Dear Editor and Reviewers,

Thank you for the opportunity to revise our manuscript, "Beyond Health: A Machine Learning Analysis of Structural Barriers to School Attainment in Somalia" (formerly "Uncovering the Health-Education Nexus..."). We are very grateful to you and the reviewers for the insightful and constructive feedback. The comments have been invaluable in helping us strengthen the manuscript’s clarity, rigor, and impact.

We have thoroughly revised the paper to address every point raised. We believe the manuscript is now significantly improved and more suitable for publication in PLOS ONE. Below, we provide a point-by-point response to each of the comments.

**Response to Editor and Reviewer Comments**

**1. General Submission Requirements**

Comment: General instructions on PLOS ONE submission requirements (rebuttal letter, track changes, formatting, etc.).

Response: We thank the editor for the clear instructions. We have ensured that our resubmission package includes all required documents: this point-by-point response letter, a revised manuscript with tracked changes, and a clean, unmarked version of the final manuscript. All formatting has been updated to align with PLOS ONE’s style guidelines.

**2. Title and Framing**

Comment: The current title, emphasizing the "health-education nexus," may mislead readers, given that the findings show structural and geographic factors are stronger predictors.

Response: This is an excellent point. We agree that the original title did not fully capture our main finding. To better reflect the core conclusion of our research, we have revised the title to: "Beyond Health: A Machine Learning Analysis of Structural Barriers to School Attainment in Somalia." This new title immediately signals that the paper’s primary focus is on the structural determinants that overshadow the direct health-education link.

**3. Abstract Clarity**

Comment: The abstract is dense with technical terms and statistical metrics, which may overwhelm some readers. The reviewer recommends simplifying and focusing the abstract.

Response: We agree that the abstract needed to be more accessible. We have revised it to focus on the core narrative of our study, reducing jargon and limiting statistical metrics to only the most essential (e.g., the top model's AUC). The revised abstract now follows a clear structure: the research gap, the main findings, and the key policy implications, making it more concise and impactful.

**4. Introduction and Literature Review**

Comment (Scope & Theoretical Application): The literature review is comprehensive but can be difficult to follow. The reviewer suggests streamlining it by grouping theories into thematic clusters and clarifying the link between the theoretical frameworks and the study's design, possibly with a conceptual diagram.

Response: We thank the reviewer for this valuable feedback. We have substantially restructured the Introduction and Literature Review to improve its flow and clarity.

Streamlining: We have condensed the literature review by explicitly grouping sources into thematic clusters, such as "Systemic Fragility in Health and Education" and "A Multi-Layered Problem: Integrating Theoretical Frameworks."

Conceptual Diagram: Following the reviewer's superb suggestion, we have created a new conceptual diagram (now Figure 1) based on Bronfenbrenner's Ecological Systems Theory. This figure visually maps our multi-level theoretical framework and illustrates how it directly informed our variable selection and analytical approach. We have added text in the Introduction to explicitly reference this new figure.

**5. Methodology - Justification for Machine Learning Approach**

Comment: The rationale for selecting the ten specific algorithms and for ultimately choosing the Random Forest model for feature importance analysis could be elaborated.

Response: We agree that this justification is crucial for methodological rigor. We have added a new paragraph in the "Statistical Analysis" section to clarify our approach. We now explain that the ten models were deliberately chosen to represent a diverse suite of algorithmic families for a robust comparison. We also explicitly state that the Random Forest model was selected for the final analysis due to its superior overall performance combined with its inherent ability to generate interpretable feature importance scores, offering an excellent balance between predictive accuracy and explainability.

**6. Methodology - Transparency**

Comment: A brief summary of preprocessing steps (e.g., treatment of missing data, variable encoding, model tuning) is needed to enhance reproducibility.

Response: We agree completely that these details are essential. This was a critical omission in our original draft. We have added a new subsection titled "Data Preprocessing and Model Training" to the Methodology section. This new subsection now explicitly details our method for k-NN imputation for missing data, our use of one-hot encoding for categorical variables, and our approach to model training using default hyperparameters, with a clear justification for this choice.

**7. Results - Interpretation and Presentation**

Comment: The paper could benefit from a more nuanced interpretation of mid- and lower-ranked predictors (especially health variables) and more visual representation. In-text references to tables and figures also needed to be clearer.

Response: We thank the reviewer for these suggestions to improve clarity.

Nuanced Interpretation: To provide a more nuanced interpretation, we have significantly expanded the Discussion section. We now dedicate a paragraph to explaining that the lower predictive power of health variables does not imply they are unimportant, but rather reflects a context of systemic health system collapse where poor health becomes a pervasive baseline condition, thus making it a poor differentiator.

Visual Representation & Consistency: We have meticulously proofread the entire manuscript to correct all in-text table and figure citations.

**8. Policy Implications - Relevance and Concreteness**

Comment: The policy implications could be made more concrete, with specific examples of interventions.

Response: This is an excellent recommendation to make our policy implications more actionable. We have revised the "Policy Implications" section to include concrete examples directly linked to our findings, such as "deploying mobile education units for nomadic populations," "integrating menstrual health support directly in schools," and focusing resources on specific "coldspot" regions like Gedo and Middle Shabelle.

**9. Style, Grammar, and Formatting**

Comment: There are some typographical errors and formatting inconsistencies throughout the manuscript.

Response: We sincerely apologize for these errors. We have conducted a thorough copy-edit and proofread of the entire manuscript to correct all typographical, grammatical, and formatting inconsistencies, including the specific examples noted by the reviewer. We have also ensured paragraphs are of a more readable length.

**10. Overall Assessment**

Comment: The reviewer notes this is an important and original study and looks forward to a revised version.

Response: We are very encouraged by the reviewer's positive assessment and appreciate the guidance provided to improve our work.

We thank the editor and the reviewers once again for their time and expertise. We believe that the revisions have substantially improved the manuscript and hope that it is now acceptable for publication in PLOS ONE.

Sincerely,

Hodo Abdikarim

Corresponding Author, on behalf of all authors
